# Supplementary material for: Competitive Coordination-Oriented Monodispersed Cobalt Sites on a N-Rich Porous Carbon Microsphere Catalyst for High-Performance Zn−Air Batteries
Source: Nanomaterials (Basel). 2023 Apr 10;13(8):1330. doi: 10.3390/nano13081330 (PMC10142557; doi:10.3390/nano13081330)
Supplement: Supplementary file 1 [file nanomaterials-13-01330-s001.zip › nanomaterials-2312932-supplementary.docx]

Supplementary Materials

**Competitive Coordination-Oriented Monodispersed Cobalt Sites on a N-Rich Porous Carbon Microsphere Catalyst for High-Performance Zn−Air Batteries**


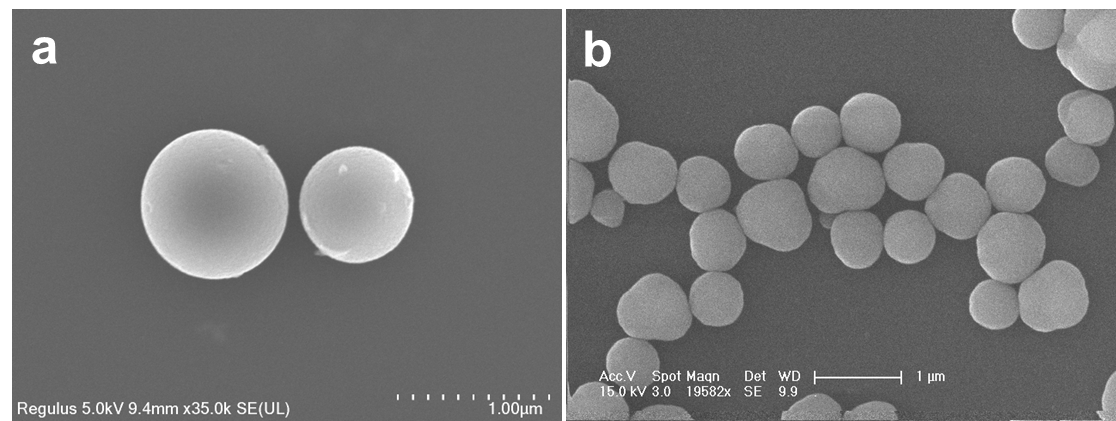


**Figure S1.** (a) SEM image of Ad−Zn SCMS. (b) SEM image of Ad−Co SCMS.


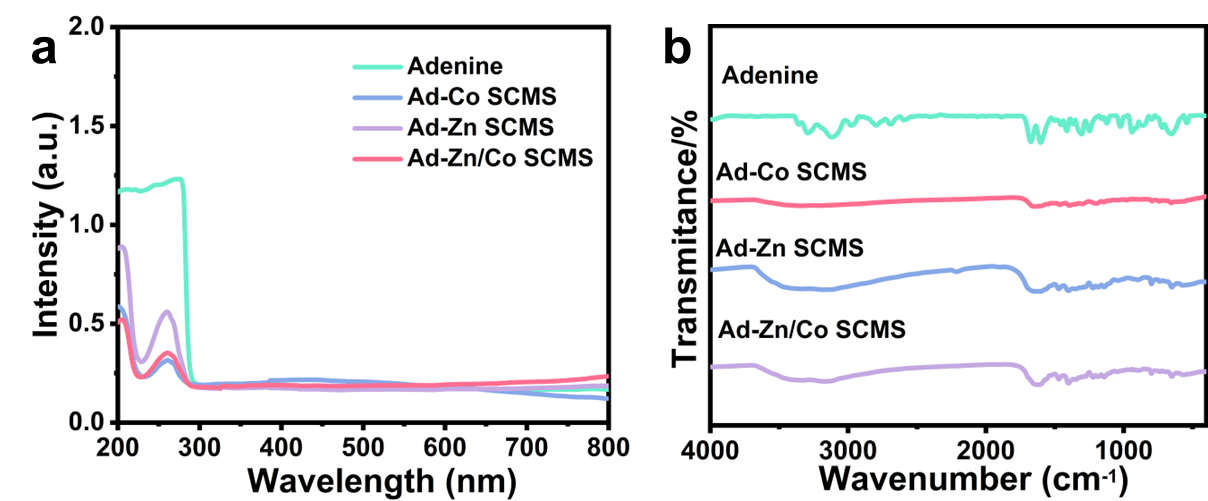


**Figure S2.** (a) UV-vis absorption of Adenine, Ad−Co SCMS, Ad−Zn SCMS and Ad−Zn/Co SCMS. (b) FTIR spectra of Adenine, Ad−Co SCMS, Ad−Zn SCMS and Ad−Zn/Co SCMS.

**
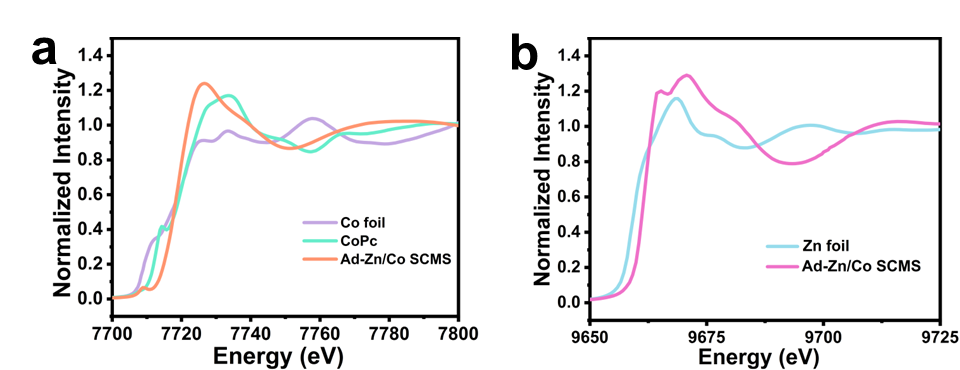
**

**Figure S3.** (a) Co K-edge XANES spectra of Co foil, CoPc and Ad−Zn/Co SCMS. (b) Zn K-edge XANES spectra of Zn foil and Ad−Zn/Co SCMS.


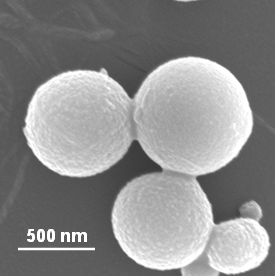


**Figure S4.** SEM image of CoSA/N−PCMS.


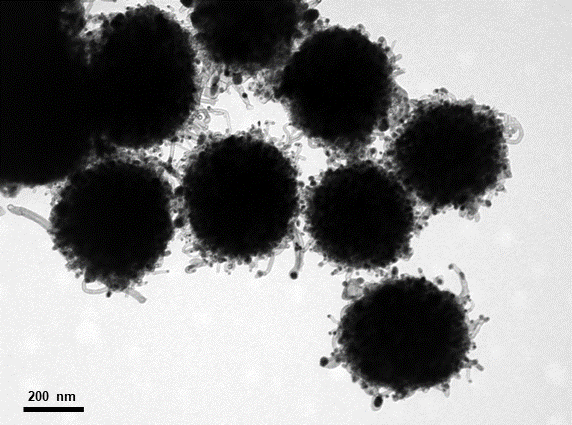


**Figure S5.** TEM image of Co/N−PCMS.


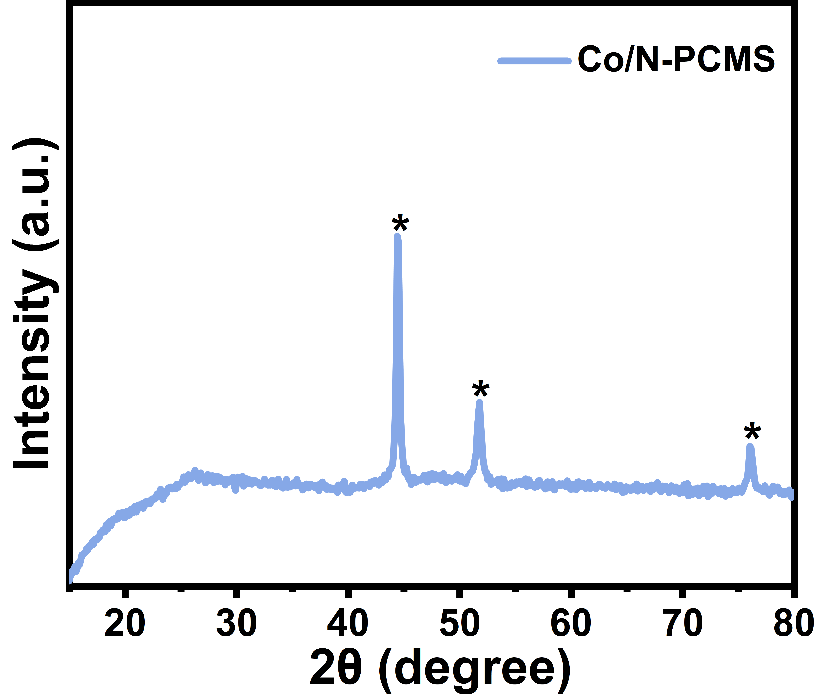


**Figure S6.** XRD pattern of Co/N−PCMS.


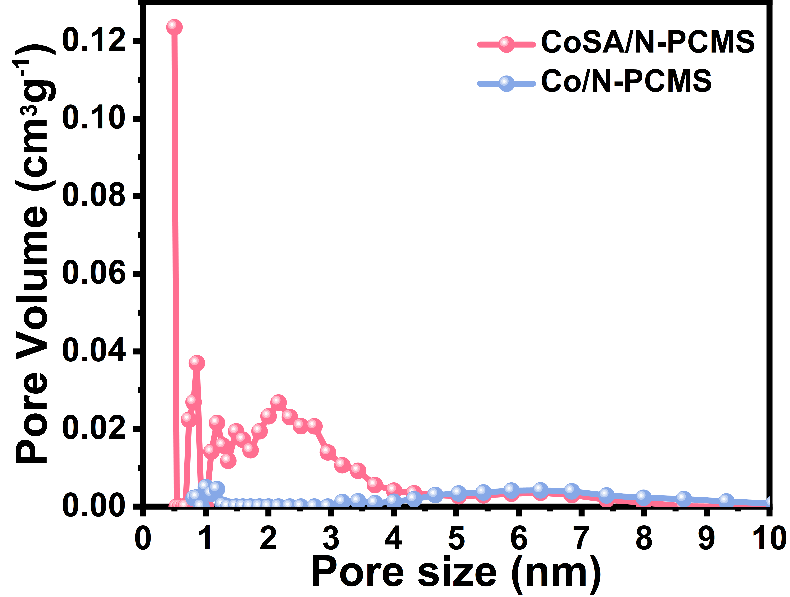


**Figure S7.** Pore size distribution curves of CoSA/N−PCMS and Co/N−PCMS.


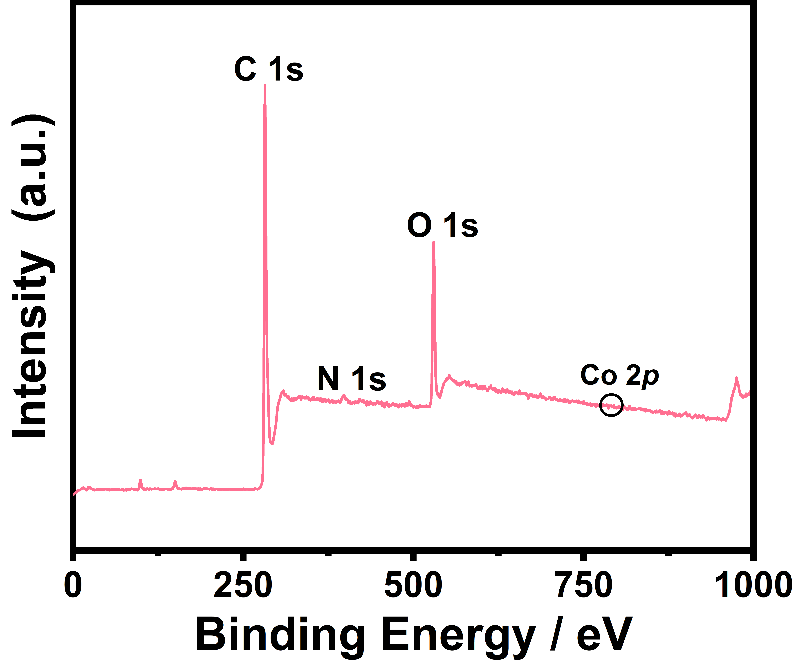


**Figure S8.** XPS full scan of CoSA/N−PCMS.


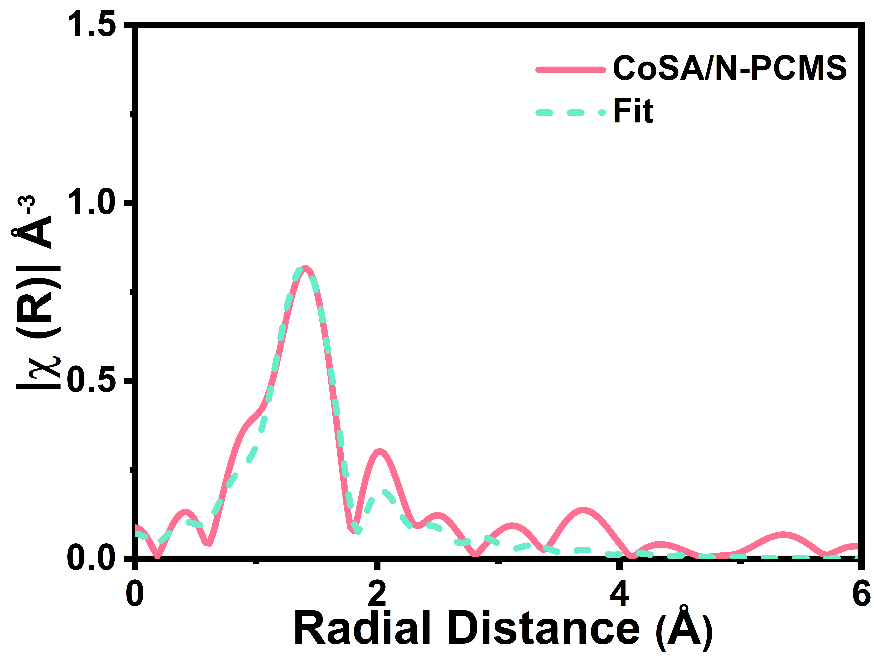


**Figure S9.** Co K-edge EXAFS fitting analysis of CoSA/N−PCMS in R space.


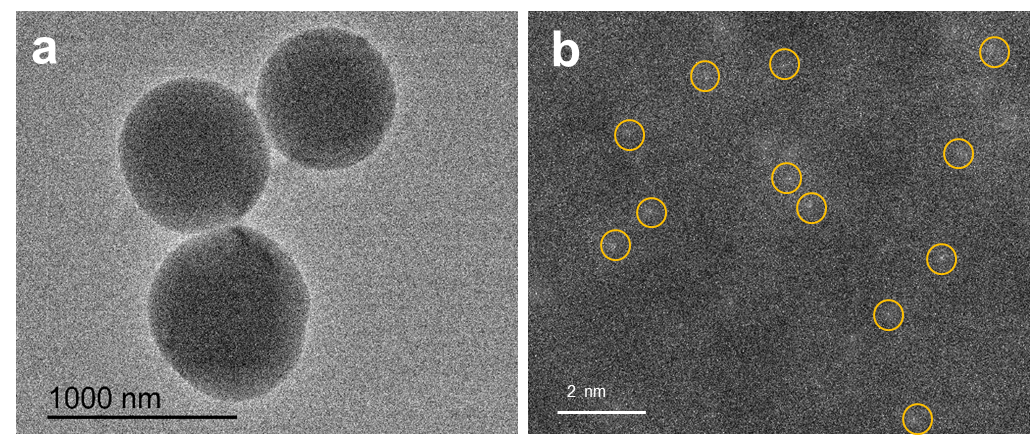


**Figure S10.** (a) TEM image of FeSA−N PCMS. (b) HAADF-STEM image of FeSA−N PCMS with partially atomically dispersed Fe atoms circled.


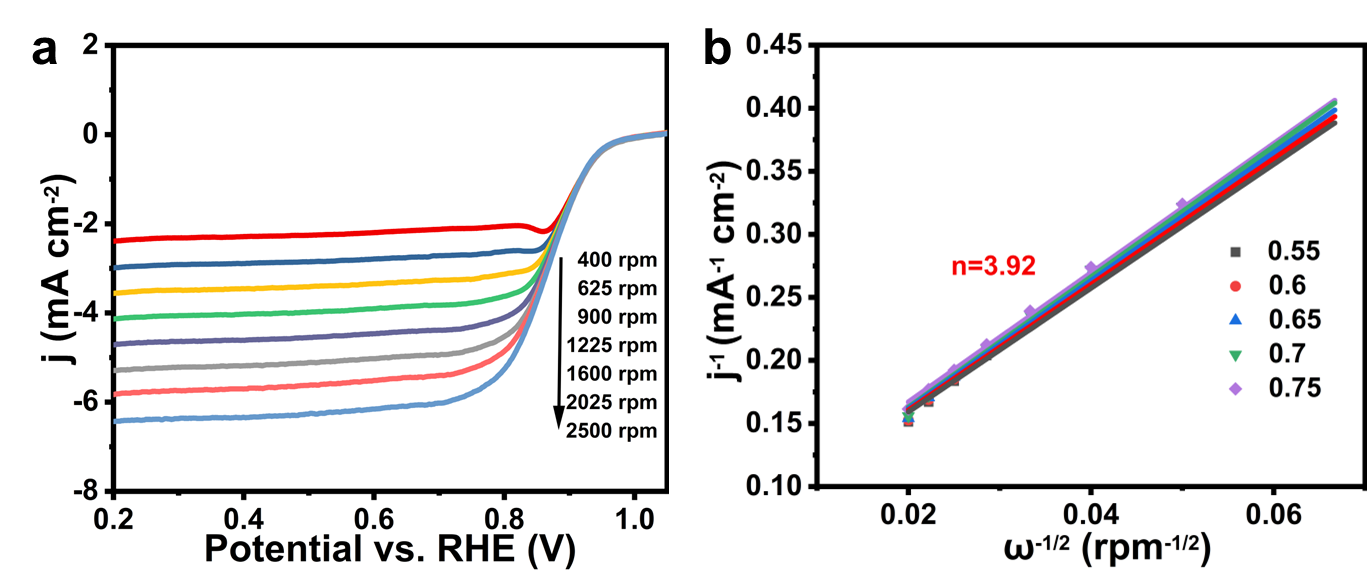


**Figure S11.** (a) LSV curves at different rotating rates of CoSA/N−PCMS. (b) the Koutecky-Levich plots.


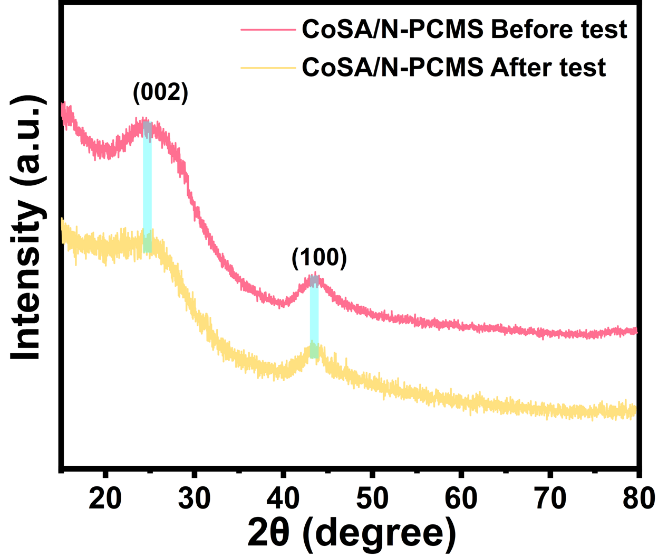


**Figure S12.** XRD patterns of CoSA/N−PCMS before and after ADT testing.


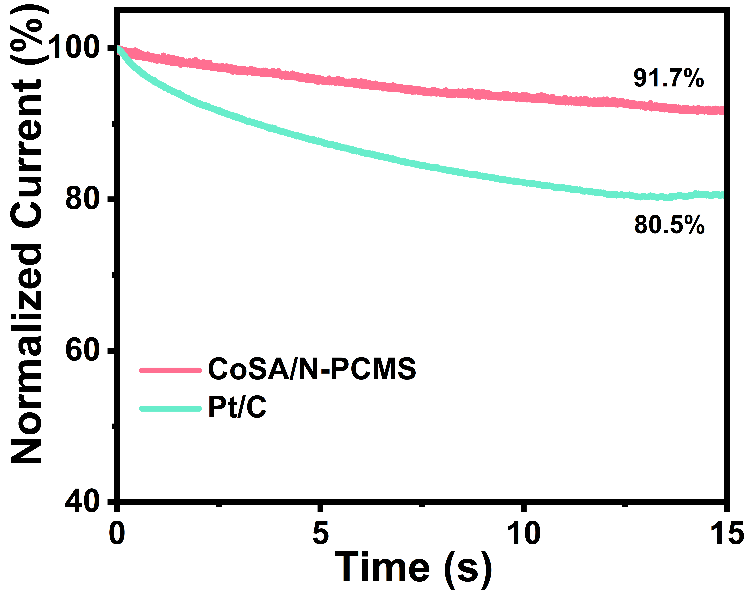


**Figure S13.** Chronoamperometry (i-t) measurement of CoSA/N−PCMS and Pt/C with a rotation rate of 1600 rpm in O_2_-saturated 0.1 M KOH.


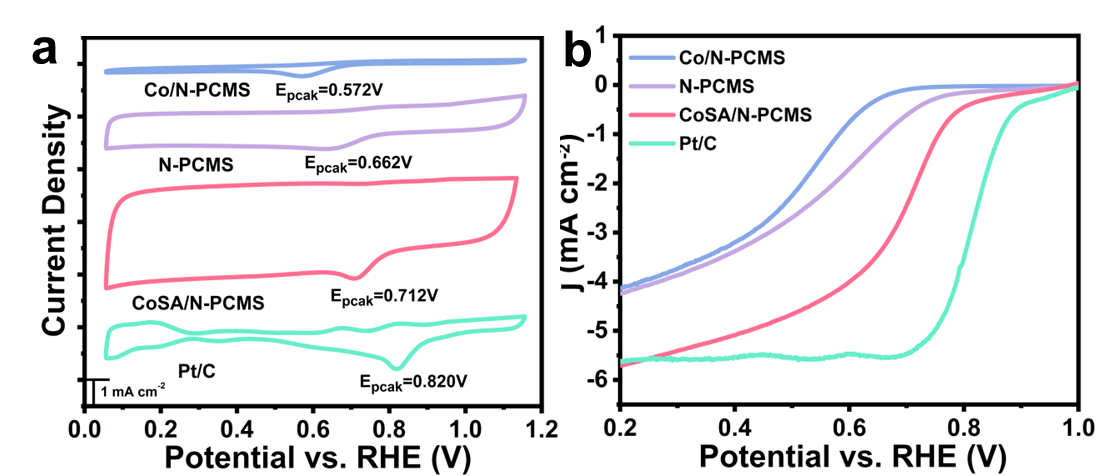


**Figure S14.** (a) CV curves of CoSA/N−PCMS, Co/N−PCMS, N−PCMS and Pt/C in O_2_-saturated 0.1 M HClO_4_ with a sweep rate of 10 mV s^−1^. (b) LSV curves of CoSA/N−PCMS, Co/N−PCMS, N−PCMS and Pt/C in O_2_-saturated 0.1 M HClO_4_ with a sweep rate of 10 mV s^−1^ and 1600 rpm.

**
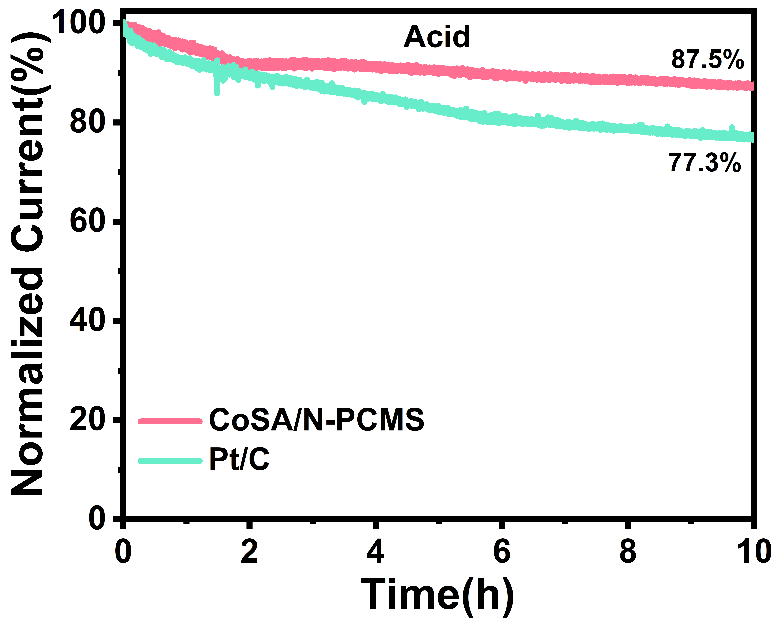
**

**Figure S15.** Chronoamperometry (i-t) measurement of CoSA/N−PCMS and Pt/C with a rotation rate of 1600 rpm in O_2_-saturated 0.1 M HClO_4_.

CV and LSV curves for acidic conditions (HClO_4_) are shown in Figure. S14a and S14b. CoSA/N−PCMS has a more obvious reduction peak compared to Co/N−PCMS and N−PCMS, and its E_onest_ (0.854 V) and E_1/2_ (0.697 V) are better than those of Co/N−PCMS and N−PCMS. However, it is slightly inferior to Pt/C. However, the stability of CoSA/N−PCMS (87.5%) was more stable than that of Pt/C (77.0%) under acidic conditions for 10 hours (Figure. S15).

**Table S1.** eZAF Smart Quant Results of CoSA/N−PCMS.

**Table S2.** EXAFS fitting parameters at the Co K-edge (S_0_^2^=0.76).

CN is the coordination number; R is the interatomic distance (the bond length between Co atom and surrounding coordinated N atoms); σ^2^ is Debye-Waller factor (a measure of thermal and static disorder in absorber-scatterer distances). R factor is used to value the goodness of the fitting.

**Table S3.** Summary of ORR activity of different catalysts in this work under alkaline condition.

Sample

E

onset

(V

vs.

RHE)

E

1/2

(V

vs.

RHE)

CoSA/N-PCMS

0.99

0.87

Co/N-PCMS

0.93

0.83

N-PCMS

0.96

0.79

Pt/C

0.98

0.83

**Table S4.** CoSA/N−PCMS compared with recently reported Co SACs.

Catalyst

E

onset

(

V vs RHE)

E

1/2

(V vs RHE)

N content

Refs

Co

SA

/N-PCMS

0.99

0.87

18.49

at%

This work

Co SA/N

-

CNS

-

900

1.00

0.87

9.

0

0

at%

[62]

CoSAs@CNTs

0.99

0.86

3.17

at

%

[63]

20Co

-

NC

-

1100

0.93

0.80

3.6

0

at%

[64]

Co/N

-

HCOs

0.92

0.81

5.08 wt%

[65]

Co

-

N

-

C

-

900

0.9

3

0.88

5.39 at%

[66]

Co

-

N

-

C SAC

1.01

0.85

10.74 wt%

[34]

References

1. Wang, L.J.; Xu, Z.X.; Peng, T.Y.; Liu, M.S.; Zhang, L.; Zhang, J.M. Bifunctional Single-Atom Cobalt Electrocatalysts with Dense Active Sites Prepared via a Silica Xerogel Strategy for Rechargeable Zinc-Air Batteries. *Nanomaterials* **2022**, *12*, 381. https://doi.org/10.3390/nano12030381.
2. Shen, T.; Huang, X.X.; Xi, S.B.; Li, W.; Sun, S.N.; Hou, Y.L. The ORR electron transfer kinetics control via Co−N-x and graphitic N sites in cobalt single atom catalysts in alkaline and acidic media. *J. Energy Chem*. **2022**, *68*, 184−194. https://doi.org/10.1016/j.jechem.2021.10.027.
3. Dilpazir, S.; He, H.; Li, Z.; Wang, M.; Lu, P.; Liu, R.; Xie, Z.; Gao, D.; Zhang, G. Cobalt Single Atoms Immobilized N-Doped Carbon Nanotubes for Enhanced Bifunctional Catalysis toward Oxygen Reduction and Oxygen Evolution Reactions. *ACS Appl. Energy Mater.* **2018**, *1*, 3283−3291. https://doi.org/10.1021/acsaem.8b00490.
4. Wang, X.X.; Cullen, D.A.; Pan, Y.T.; Hwang, S.; Wang, M.; Feng, Z.; Wang, J.; Engelhard, M.H.; Zhang, H.; He, Y.; et al. NitrogeN−Coordinated Single Cobalt Atom Catalysts for Oxygen Reduction in Proton Exchange Membrane Fuel Cells. *Adv Mater* **2018**, *30*, 1706758. https://doi.org/10.1002/adma.201706758.
5. Chao, S.; Bai, Z.; Cui, Q.; Yan, H.; Wang, K.; Yang, L. Hollowed-out octahedral Co/N−Codoped carbon as a highly efficient noN−Precious metal catalyst for oxygen reduction reaction. *Carbon* **2015**, *82*, 77−86. https://doi.org/10.1016/j.carbon.2014.10.034.
6. Zhu, C.; Shi, Q.; Xu, B.Z.; Fu, S.; Wan, G.; Yang, C.; Yao, S.; Song, J.; Zhou, H.; Du, D.; et al. Hierarchically Porous M−N−C (M = Co and Fe) Single‐Atom Electrocatalysts with Robust MNx Active Moieties Enable Enhanced ORR Performance. *Adv. Energy Mater.* **2018**, *8*, 1801956. https://doi.org/10.1002/aenm.201801956.
